# Unraveling the unfolded protein response signature: implications for tumor immune microenvironment heterogeneity and clinical prognosis in stomach cancer

**DOI:** 10.18632/aging.205784

**Published:** 2024-05-02

**Authors:** Wenhao Ouyang, Yajing Liu, Hong Huang, Yujing Tan, Zhenjun Huang, Xueyuan Jia, Yunfang Yu, Herui Yao

**Affiliations:** 1 Guangdong Provincial Key Laboratory of Malignant Tumor Epigenetics and Gene Regulation, Department of Medical Oncology, Breast Tumor Centre, Phase I Clinical Trial Centre, Yat-Sen Supercomputer Intelligent Medical Joint Research Institute, Sun Yat-Sen Memorial Hospital, Sun Yat-Sen University, Guangzhou 510120, Guangdong, China; 2 School of Medicine, Guilin Medical University, Guilin 541000, Guangxi, China; 3 Department of Radiation Oncology, Zhujiang Hospital, Southern Medical University, Guangzhou 510515, Guangdong, China; 4 Faculty of Medicine, Macau University of Science and Technology, Taipa 999078, Macao, P.R. China

**Keywords:** stomach cancer, unfolded protein response, prognostic signature, IGFBP1, tumor immune microenvironment

## Abstract

Background: Stomach cancer is a leading cause of cancer-related deaths globally due to its high grade and poor response to treatment. Understanding the molecular network driving the rapid progression of stomach cancer is crucial for improving patient outcomes.

Methods: This study aimed to investigate the role of unfolded protein response (UPR) related genes in stomach cancer and their potential as prognostic biomarkers. RNA expression data and clinical follow-up information were obtained from the TCGA and GEO databases. An unsupervised clustering algorithm was used to identify UPR genomic subtypes in stomach cancer. Functional enrichment analysis, immune landscape analysis, and chemotherapy benefit prediction were conducted for each subtype. A prognostic model based on UPR-related genes was developed and validated using LASSO-Cox regression, and a multivariate nomogram was created. Key gene expression analyses in pan-cancer and *in vitro* experiments were performed to further investigate the role of the identified genes in cancer progression.

Results: A total of 375 stomach cancer patients were included in this study. Analysis of 113 UPR-related genes revealed their close functional correlation and significant enrichment in protein modification, transport, and RNA degradation pathways. Unsupervised clustering identified two molecular subtypes with significant differences in prognosis and gene expression profiles. Immune landscape analysis showed that UPR may influence the composition of the tumor immune microenvironment. Chemotherapy sensitivity analysis indicated that patients in the C2 molecular subtype were more responsive to chemotherapy compared to those in the C1 molecular subtype. A prognostic signature consisting of seven UPR-related genes was constructed and validated, and an independent prognostic nomogram was developed. The gene IGFBP1, which had the highest weight coefficient in the prognostic signature, was found to promote the malignant phenotype of stomach cancer cells, suggesting its potential as a therapeutic target.

Conclusions: The study developed a UPR-related gene classifier and risk signature for predicting survival in stomach cancer, identifying IGFBP1 as a key factor promoting the disease’s malignancy and a potential therapeutic target. IGFBP1’s role in enhancing cancer cell adaptation to endoplasmic reticulum stress suggests its importance in stomach cancer prognosis and treatment.

## INTRODUCTION

Stomach cancer poses a significant health burden, with low survival rates, especially among patients with metastatic disease [1]. The efficacy of current treatment options, including surgery, chemotherapy, and targeted therapies, is hindered by the intricate nature of cancer and the dynamic immune landscape [2, 3]. Enhancing patient prognosis requires the development of innovative prognostic models and exploration of new therapeutic approaches to aid clinical decision-making and improve outcomes.

The unfolded protein response (UPR) is a cellular stress response activated by unfolded or misfolded proteins accumulated in the endoplasmic reticulum (ER) that overwhelm ER’s coping ability [4, 5]. The unfolded protein response (UPR) is known to induce cell cycle arrest, activate transcription of UPR-related stress genes, inhibit protein translation, and facilitate the degradation of misfolded proteins through the activation of specific kinases. In cases where unresolved endoplasmic reticulum (ER) stress persists, the UPR may shift towards promoting apoptosis [6–8]. As the UPR maintains ER homeostasis and regulates autophagy, glucose metabolism, redox balance and so on, the impact of UPR on malignant tumor is complicated. Accumulating studies indicated the prognostic value of UPR [9, 10]. In X-box binding protein 1 (XBP1)-luciferase transgenic mice, variations in UPR reflected different metabolic and hypoxic microenvironment and predicted tumor growth [11]. In cutaneous melanoma, hepatocellular carcinoma, bladder cancer, breast cancer and glioma, UPR related signature has been constructed to predict survival or therapeutic effect [12–16]. As tumor cells often grow under stressful conditions, UPR activation affects cell fate decisions and is reasonably believed to be a prognostic marker.

In this study, UPR-related gene expression was utilized to stratify patients with stomach cancer into two molecular subtypes, which demonstrated contrasting survival rates, immune characteristics, chemotherapy responses, and stemness scores. A prognostic signature based on UPR-associated genes was developed to predict patient outcomes, and a nomogram was created to assess the risk level. These findings underscore the potential of UPR-related genes in identifying high-risk patients, facilitating personalized treatment approaches, and pinpoint IGFBP1 as a promising target for therapeutic intervention.

## MATERIALS AND METHODS

### The acquisition of clinical and gene expression data

The latest RNA expression data and clinical follow-up information were downloaded from The Cancer Genome Atlas (TCGA) and Gene Expression Omnibus (GEO) database. To eliminate batch effects between datasets, data correction was performed through the combat algorithm. Then, the HALLMARK UNFOLDED PROTEIN RESPONSE related genes were acquired from the Molecular Signatures Database (https://www.gsea-msigdb.org/gsea/msigdb/index.jsp). These genes have been found to have an important role during the unfolded protein response process in previous studies.

### Genotyping of unfolded protein response in stomach cancer

Clustering unsupervised analysis was used to identify genotyping from the expression of UPR-related genes. Then, patients in each genotyping were performed to survival analysis. The number of clusters and stability of clusters were determined by consensus clustering algorithm. Using R package “ConsensuClusterPlus”, 100 iterations were performed to ensure classification stability with the above steps.

### Analysis of functional enrichment

The UPR genomic subtype of stomach cancer was identified through unsupervised clustering analysis. Differential gene analysis was subsequently conducted using the “limma” package, comparing cluster 1 molecular subtype and cluster 2 molecular subtype with the criteria of log_2_ fold change (FC) > 1 or < -1, and a false discovery rate (FDR) p-value below 0.05. Concurrently, Gene Ontology (GO) functional analysis and Kyoto Encyclopedia of Genes and Genomes (KEGG) pathway enrichment analysis were performed based on the differentially expressed genes (DEGs). The “clusterProfiler” R package (v3.0.0) was employed for KEGG pathway analysis and Gene Ontology (GO) analysis.

### The immune landscape analysis

The Extended Polydimensional Immunome Characterization (EPIC), quanTIseq (a method for quantifying tumor immune contexture), xCell, and MCPcount were employed to evaluate cellular components and immune responses between clusters based on UPR-related genes. The purpose of this comparison was to thoroughly assess the stomach cancer immune microenvironment, as a comprehensive understanding of the immune landscape can provide valuable insights into tumor progression, prognosis, and response to therapy. By examining the immune cell composition and interactions, researchers can identify potential therapeutic targets and develop more effective strategies to improve patient outcomes.

### Chemotherapy benefit predicted by clusters

In this study, we predicted the responsiveness of each sample to chemotherapeutic drugs, a process that utilized the largest publicly available drug genome database, Genomics of Drug Sensitivity in Cancer (GDSC, http://www.cancerrxgene.org/). To facilitate the prediction process, we used the R language package pRRophetic. This toolkit enables researchers to predict drug responsiveness in tumor samples based on the genomic features of drugs by integrating a large amount of drug sensitivity data and related gene expression information from the GDSC database.

During the prediction process, we first normalized the gene expression data in the GDSC database to eliminate the differences between the data under different experimental conditions and ensure the accuracy of the prediction. Subsequently, pRRophetic estimated the half-maximal inhibitory concentration (IC_50_) of each sample by a ridge regression (Ridge Regression) algorithm. Ridge regression is an improved linear regression method that deals with the problem of multicollinearity among variables by introducing a regularization term to enhance the predictive stability and accuracy of the model.

### Developing and validating prognostic models of UPR-related signature

The number of UPR-related genes was reduced using LASSO-Cox regression. Individual normalized gene expression levels, weighted by their corresponding multiple-Cox coefficients, were utilized to develop a UPR-related score formula. The external validation cohort was then employed to verify the stability of this prognostic formula. The log-rank test was used to assess survival differences between two groups. The AUC of ROC was calculated to evaluate the prediction model performance. The Area Under the Curve (AUC) of the Receiver Operating Characteristic (ROC) curve was computed to assess the efficacy of the prediction model in utilizing risk scores for predicting breast cancer survival rates over 1-year, 3-year, and 5-year survival [17]. The ROC analysis, conducted with the ‘timeROC’ package in R, was specifically employed for constructing the ROC curves to evaluate the predictive performance of the model.

### Develop a multivariate nomogram

Univariate Cox regression analysis was performed to screen the overall survival (OS) associated UPR-related genes involved in score formula. Next, the multivariate Cox regression analysis of the same genes with clinical characteristics identified independent factors associated with stomach cancer prognosis. Following a multivariate Cox analysis, genes or clinical characteristics with p-values less than 0.05 were considered as independent prognostic factors. Using the nomogram function provided in the ‘rms’ library, a nomogram was created in R software.

### Key gene expression analyses in pan-cancer

Pan-cancer analyses were performed on the gene with the highest correlation coefficient which was considered as the key gene. The differential expression of this gene in other pan-cancer tissues and the adjacent normal tissue, and the association of expression of this gene with ESTIMATE score and Stemness genes in tumors were analyzed for investigation of this gene’s role in cancer.

### Cells culture

The immortalized gastric epithelial cell line (GES-1) and three GC cell lines (MKN45, AGS and HGC-27) were obtained from Sun Yat-sen Memorial Hospital, Sun Yat-sen University. GES-1, MKN45 and HGC-27 cells were cultured in RPMI-1640 medium with 10% fetal bovine serum (FBS) and 1% penicillin/streptomycin, while AGS cells were cultured in F12K medium with 10% FBS and 1% penicillin/streptomycin. The culture atmosphere is 37° C, 90% humidity, and 5% CO_2_.

### Cell transfection

Before transfection, two stomach cancer cell lines, MKN45 and AGS, were transferred into 6-well plates. A combination of small interfering RNA (siRNA) (Ribobio, Guangzhou, China) mixed with Lipofectamine 2000 transfection reagent (Invitrogen, CA, USA) was used to transfect the tumor cells after they had reached a sufficient confluency. In the experiment, siRNAs that are validated were transfected into the cells, while nontarget control siRNAs were transfected into the control cells. The complete medium was added to the transfected cells after 6h of incubation with the siRNAs, and the cells were cultured for 48 h before being used for further experiments. For the construction of shRNA expression vector targeting IGFBP1, a small hairpin RNA (shRNA) containing specific sequences targeting the human IGFBP1 (sequence: 5’-GATCTGGTACTGCTCCTGCTGACTGCTCGAGCAGTCAGCAGGAGCAGTACCATTTTTG-3’) was cloned into the pSilencer 3.1-H1 puro (Thermo Fisher Scientific, MA, USA), followed by transfection into *E. coli* DH5α for propagation. Transfection of this vector into GC cell lines MKN45 established the knockdown group (shIGFBP1), with cells harboring control sequences forming the mock group (shNC). Then, for overexpression of IGFBP1, the open reading frame (ORF) of human IGFBP1 was cloned into a pcDNA3.1 vector, which was followed by transfection with Lipofectamine 3000 (Invitrogen, MA, USA).

### RNA isolation and RT-qPCR analysis

To extract total RNA, TRIzol reagent (Vazyme Biotech Co., Ltd., Nanjing, China) was utilized. According to the manufacturer’s instructions, 1 μg cDNA was generated using the HiScript III first Strand cDNA Synthesis Kit (Vazyme Biotech Co., Ltd., Nanjing, China). Then, we used the Quant Studio TMDx from Applied Biosystems (MA, USA) as well as the ChamQ Universal SYBR qPCR Master Mix kit from Vazyme Biotech (Vazyme Biotech Co., Ltd., Nanjing, China). Activation of the polymerase was performed at 95° C for 30 seconds, followed by 35 cycles at 95° C for 5 seconds and 60° C for 30 seconds. The 2^–ΔΔCt^ method was used to determine the relative expression of mRNA. Gene expression levels were normalized using β-actin.

### Cell proliferation assay

96-well culture plates with 1.0 × 10^3^ cells per well were seeded with cells and cultured for 3 days. CCK8 reagent (Dojindo Laboratories, Kumamoto, Japan) was added to each well every 24 hours and incubated for 1 hour at 37° C to determine the viable cells. The microplate reader (Molecular Devices, CA, USA) was used to measure the absorbance at 450 nm. In addition, approximately 700 cancer cells were plated into six-well plates, and the cells were allowed to grow for approximately two weeks. The mixture of 4% paraformaldehyde and 0.5% crystal violet staining was performed for 15 minutes after two washes with phosphate-buffered saline (PBS). Cell clones were calculated for different groups.

### Flow cytometry assay

The Annexin V-FITC/PI Cell Apoptosis Detection Kit (Beyotime Biotechnology, Shanghai, China) was used for flow cytometry. (FITC)-Annexin V and propidium iodide (PI) were used to stain the collected cells. BD FACSCalibur (BD, CA, USA) was used to measure and analyze the apoptosis rate of cells.

### Transwell migration and invasion assay

To assess cell invasion and migration, we utilized a Boyden chamber with Transwell membrane filter inserts (Corning, NY, USA). For the invasion assays [18, 19], Corning™ BioCoat™ Matrigel™ Invasion Chambers with Corning™ Matrigel Matrix (Thermo Fisher Scientific, MA, USA) were employed. AGS cells were suspended in 100 μL of serum-free F12K medium and added to the upper chamber, while MKN45 cells were treated similarly using serum-free RPMI-1640 medium. For both cell lines, 5×10^4 cells were used. The lower chambers were filled with 600 μL of medium containing 10% fetal bovine serum. After 24 hours for migration assays and 48 hours for invasion assays, non-migrating/non-invading cells were gently removed with a cotton swab. Cells that migrated or invaded through the membrane were fixed with 4% paraformaldehyde for 10 minutes and stained with 0.5% crystal violet for another 10 minutes. The membranes were then photographed under an inverted microscope at 200x magnification after sealing with neutral gum. Cell counting was performed using Image Pro Plus Version 6 software. In each experiment, three wells were used per group, and within each well, five random fields of view were selected for calculating the average number of cells.

### Statistical analysis

Comparing gene expression between different clusters was done using Wilcoxon rank-sum test. Kaplan-Meier analysis was used to compare survival rates among groups using the log-rank test. Cox regression analyses of univariate and multivariate models were conducted to identify independent predictor. A statistically significant *P*-value was less than 0.05. All statistical analyses were performed with R software (Version 4.0.3).

### Availability of data and materials

The data of this study will be made available by the corresponding authors.

## RESULTS

### Clinical characteristics of stomach cancer patients

**Figure 1:**
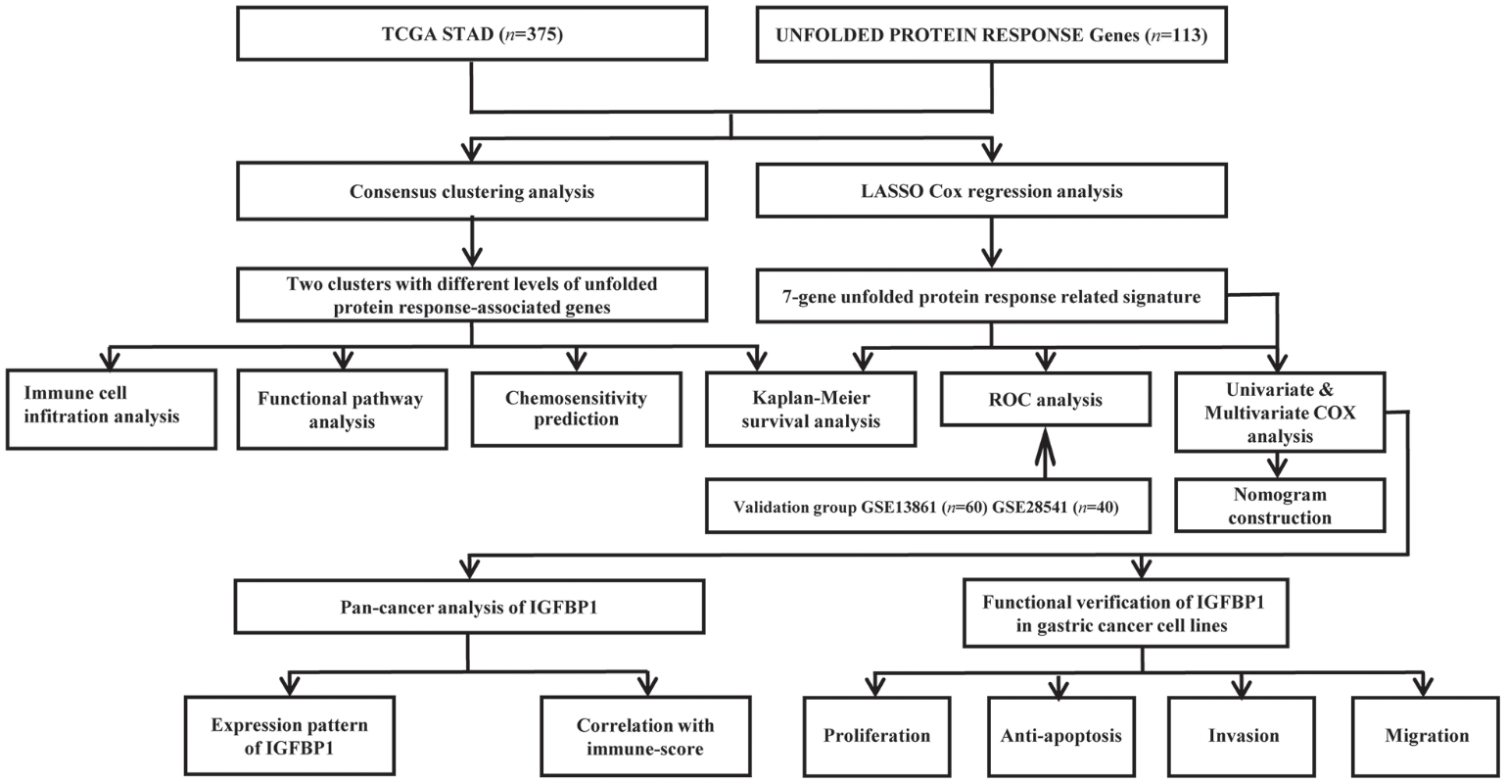
Flow chart of this study design.

A total of 375 stomach cancer patients from the TCGA cohort, 60 patients from the GSE13861 cohort, and 40 patients from the GSE28541 cohort were included in our study. The clinical characteristics of these patients are detailed in Supplementary Table 1. Additionally, we gathered 113 unfolded protein response genes. The study’s flow chart is illustrated in Figure 1.

### Expression characteristics of UPR genes in stomach cancer

**Figure 2:**
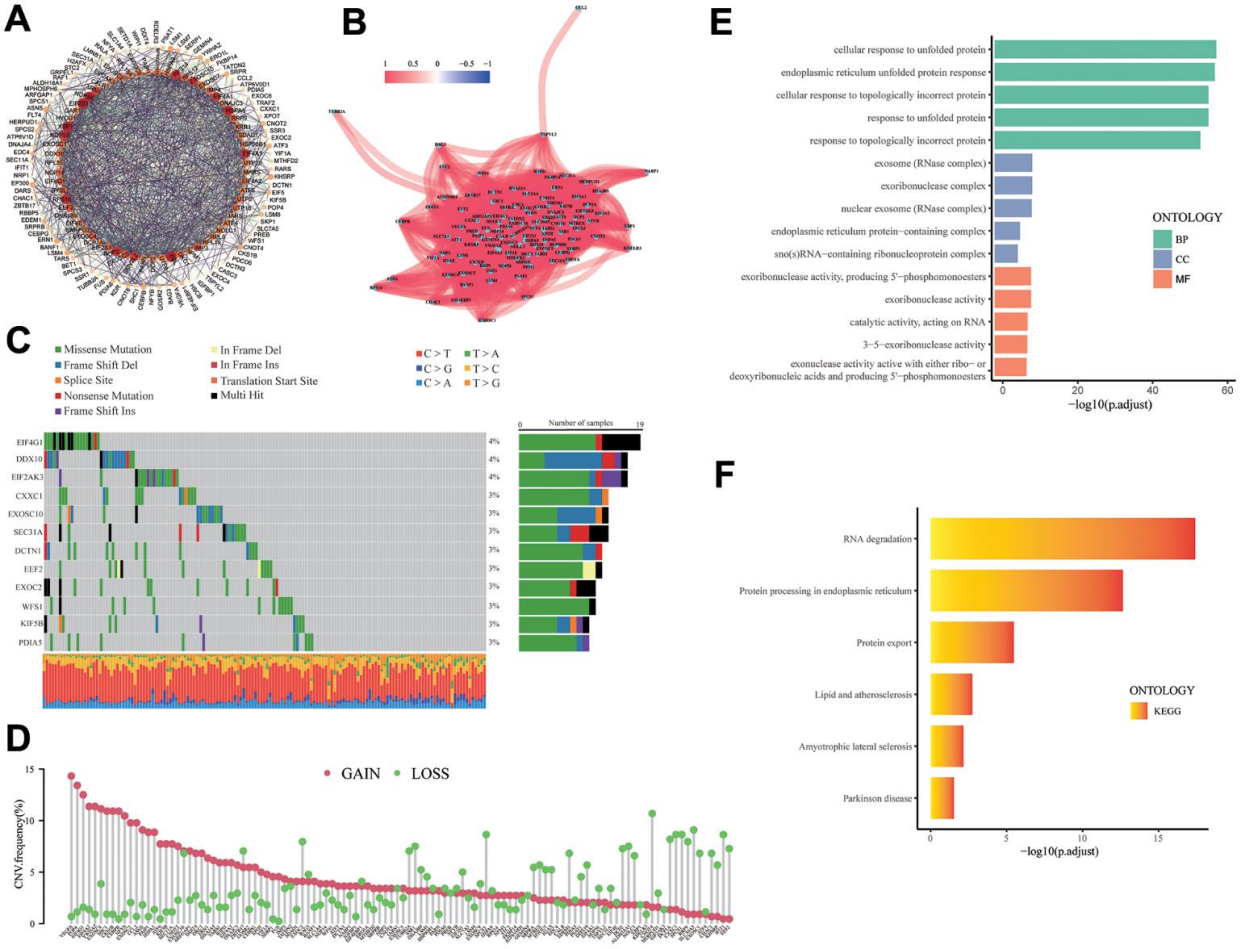
**The analysis of UPR-related genes.** (**A**) PPI network for the UPR-related genes. (**B**) Correlation network for the UPR-related genes. (**C**) Mutation analysis of UPR-related genes. (**D**) The lollipop diagram shows the CNV profile of the UPR-related genes. (**E**) GO and (**F**) KEGG pathways of the UPR-related genes.

The expression and functional connections of 113 UPR related genes were displayed with protein-protein interaction (PPI) network, and according to the correlation expression network, there is a positive correlation between the expression of UPR-related genes, demonstrating the close correlation between their functions (Figure 2A, 2B). The distribution of mutations of 133 UPR related genes in stomach cancer were exhibited in waterfall plot, it is estimated that EIF4G1, DDX10, and EIF2AK3, which have the highest mutation rate, have a mutation rate of 4%, and the mutation type is predominantly missense mutations (Figure 2C). Among all types of mutations, the missense mutation was the one with the highest frequency. Meanwhile, C>T substitution obtained the highest proportion in single nucleotide polymorphism (SNP) in stomach cancer patients. Copy number variations (CNV) analysis of 113 UPR related genes were also performed (Figure 2D). Among them, Vascular endothelial growth factor A (VEGFA) had the highest CNV gain mutation, whereas C-C motif chemokine receptor 4-NOT transcription complex subunit 4 (CNOT4) presented the most obvious deletion mutation. Gene Ontology (GO) and KEGG pathways analysis revealed that 113 UPR related genes were significantly enriched in protein modification and transport and RNA degradation (Figure 2E, 2F).

### Identification of molecular subtypes of UPR genes in stomach cancer

**Figure 3:**
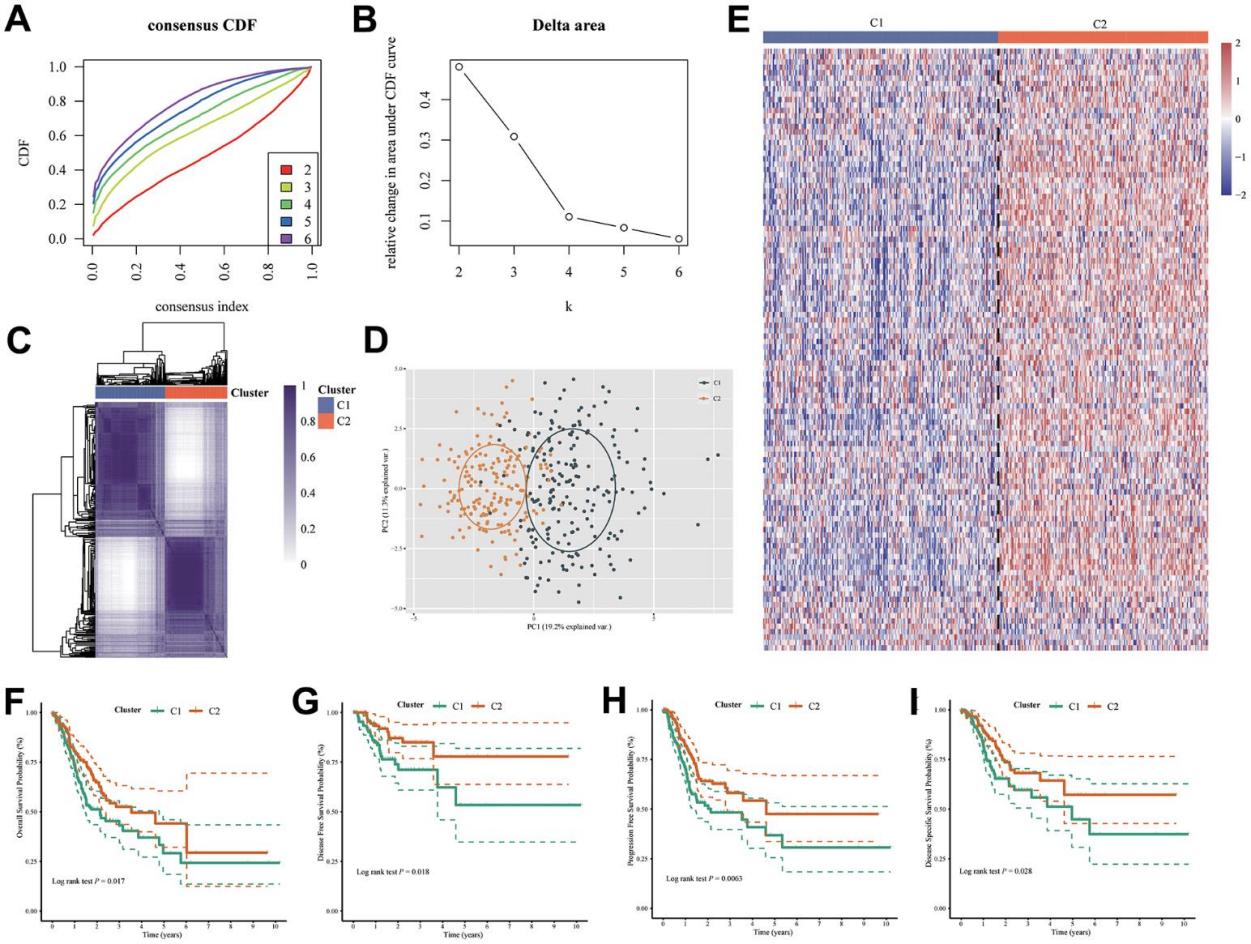
**Two molecular subtypes were identified by UPR-related gene expression.** (**A**) Consensus clustering cumulative distribution function (CDF) curve for k = 2–6. (**B**) Relative change in area under CDF curve for k = 2–6. (**C**) Consensus map, (**D**) PCA and (**E**) heatmap for UPR related gene classifier. The difference of (**F**) overall survival, (**G**) disease-free survival, (**H**) progression-free survival and (**I**) disease-specific survival between C1 and C2 group patients.

In an unsupervised Cophenetic, Silhouette Indicator analysis, two subtypes of clusters were determined to be the optimal number based on UPR-related genes in TCGA cohort (Figure 3A–3C).

Based on the principal component analysis (PCA), it was concluded that the distribution of patients clustered according to UPR-related genes was significantly different between the two types of patients (Figure 3D). Then, we showed that the expression of UPR-related genes in the two clusters were different by the heatmap (Figure 3E). Furthermore, we also analyzed the prognostic relationship between the two molecular subtypes, with the results showing that Cluster 1 (C1) group had a less favorable over-all survival (OS) probability (log rank test *P* = 0.017), disease-free survival probability (DFS) (log rank test *P* = 0.018), progression-free survival probability (PFS) (log rank test *P* = 0.0063) and disease specific survival (DSS) probability (log rank test *P* = 0.028) probability than the Cluster 2 (C2) group (Figure 3F–3I). In light of the above results, it is evident that, by clustering patients based on UPR signatures, we are able to correctly classify stomach cancer patients into molecular types, and there is indeed a difference in prognosis between them.

### Differentially analysis of GO and KEGG pathways

**Figure 4:**
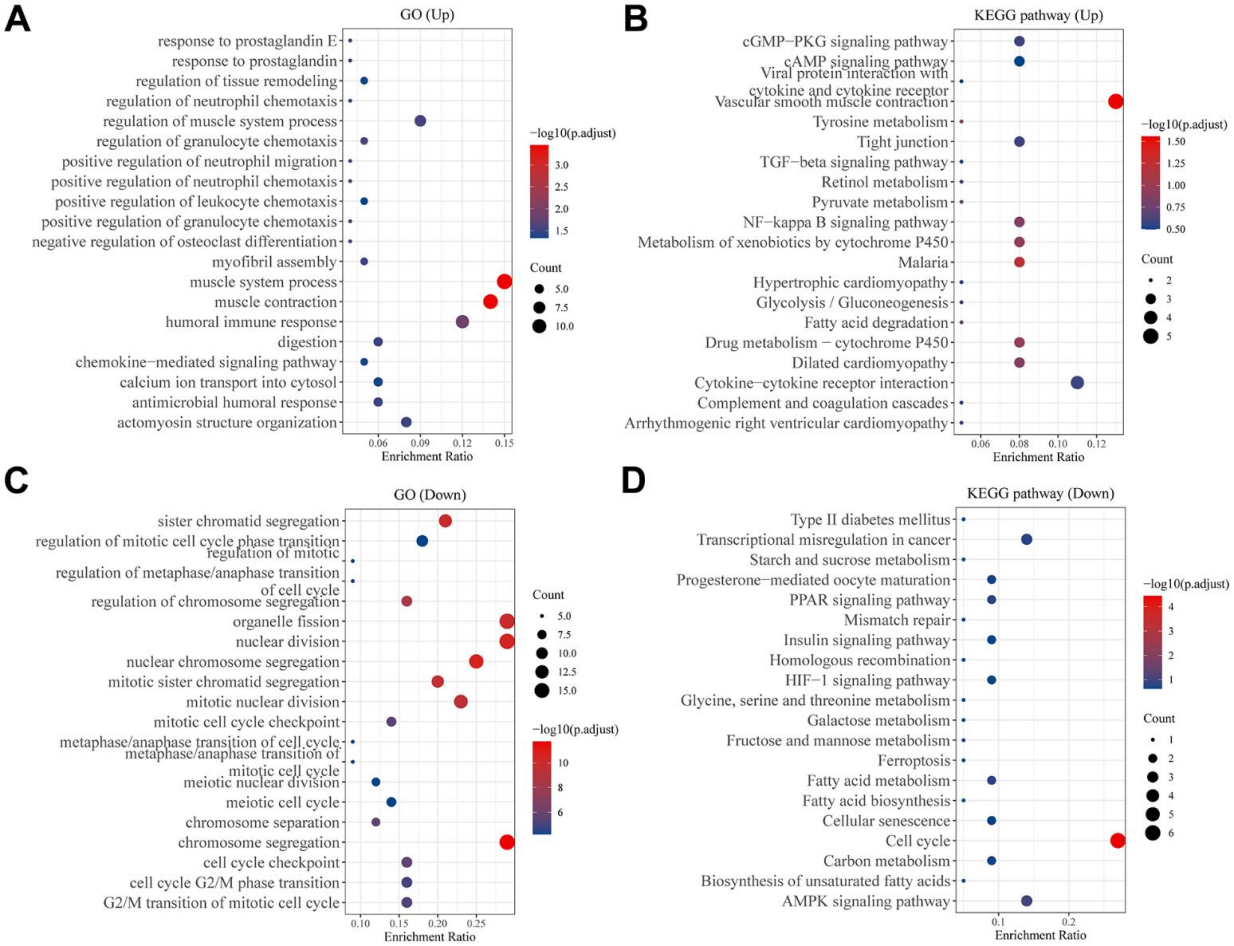
**Analysis of differentially expressed genes between two subtypes.** Bubble plot of (**A**) GO and (**B**) KEGG pathway enrichment analysis of up-regulated genes in C1 group patients. Bubble plot of (**C**) GO and (**D**) KEGG pathway enrichment analysis of down-regulated genes in C1 group patients. Each graph showed the top twenty enriched pathways.

The DEGs between C1 and C2 molecular subtypes can be calculated via the “limma” package, in which 84 genes in C1 that are up-regulated and 57 genes in C1 molecular subtype that are down-regulated. Then, GO and KEGG analysis results showed that 84 up-regulated genes in the C1 molecular subtype were significantly enriched in some cancer progressed biological processes and pathways, such as TGF-β signaling pathway, NF-κB signaling pathway (Figure 4A, 4B), in contrast, 57 down-regulated genes are enriched in the ferroptosis, adenosine 5’-monophosphate (AMP)-activated protein kinase (AMPK) signaling pathway and so on (Figure 4C, 4D).

### Differentially analysis of immune landscape

**Figure 5:**
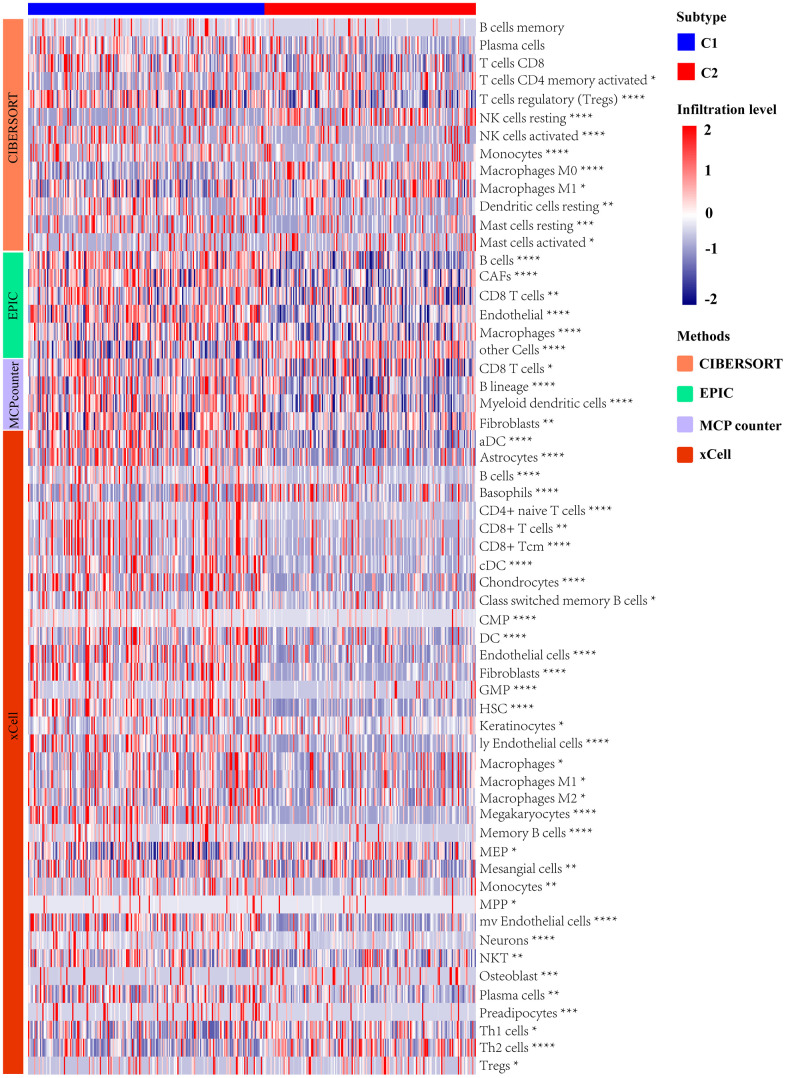
Analysis of immune landscape between two subtypes distinguished by UPR-related gene expression.

We utilized multiple algorithms for comprehensive immune cell abundance assessment in order to clarify differences between the molecular subtypes of immune cell infiltration, including CIBERSORT, EPIC, MCPcounter, and xCell. Types of tumor-infiltrating immune cells were profiled by the heatmap (Figure 5). The proportion of B cells, CD8^+^ T cells, and macrophages were significantly higher in C1 molecular subtype with lower UPR-related genes expression than C2 molecular subtype. This result suggested that the unfolded protein response may affect the composition of the tumor immune microenvironment.

### Comparative analysis of drug sensitivity

**Figure 6:**
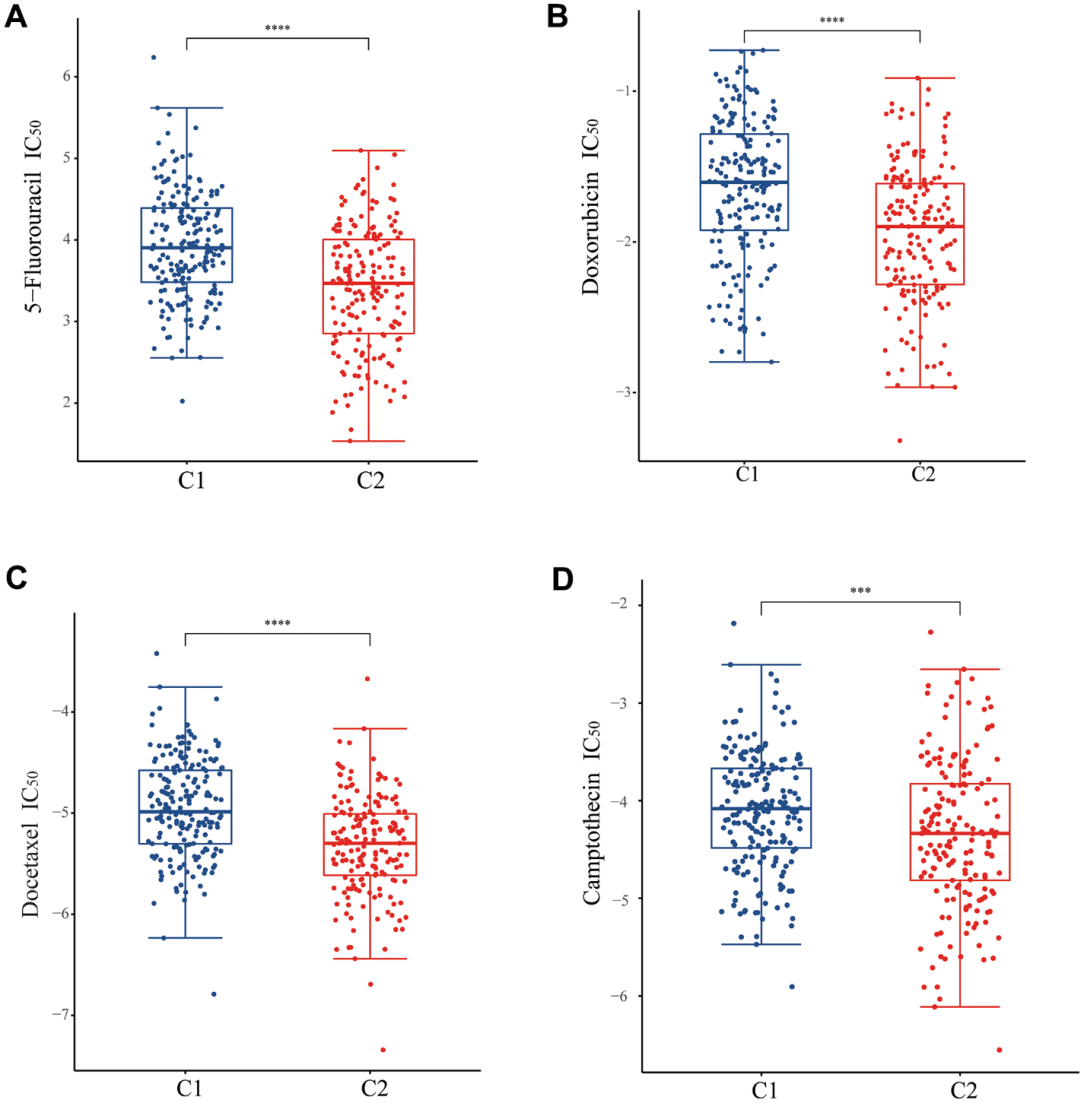
**Analysis of drug sensitivity between two subtypes.** The difference of IC_50_ of (**A**) 5-fluorouracil, (**B**) doxorubicin, (**C**) docetaxel, and (**D**) camptothecin between two subtypes.

For the purpose of understanding the sensitivity of patients with C1 and C2 molecular subtypes to chemotherapy, we examined the sensitivity of chemotherapy drugs to two different groups of patients. The IC_50_ for 5-fluorouracil, doxorubicin, docetaxel, and camptothecin was lower in patients with C2 molecular subtype, indicating an increased sensitivity to chemotherapy in these patients. (Figure 6A–6D). In conclusion, the patients in C2 molecular subtype are indeed more sensitive to chemotherapy than those in C1 molecular subtype. Previously, survival analyses have shown that patients in C2 molecular subtype have a better prognosis than patients in C1 molecular subtype, the UPR-related classifier distinguished different prognosis of patients with stomach cancer via the potential influence on the sensitivity of chemotherapy drugs. Consequently, UPR-related gene function could be correlated with treatment sensitivity in stomach cancer patients, and UPR-signature related typing could be used as a reference index for treatment selection in stomach cancer patients.

### Prognostic signature construction and validation

**Figure 7:**
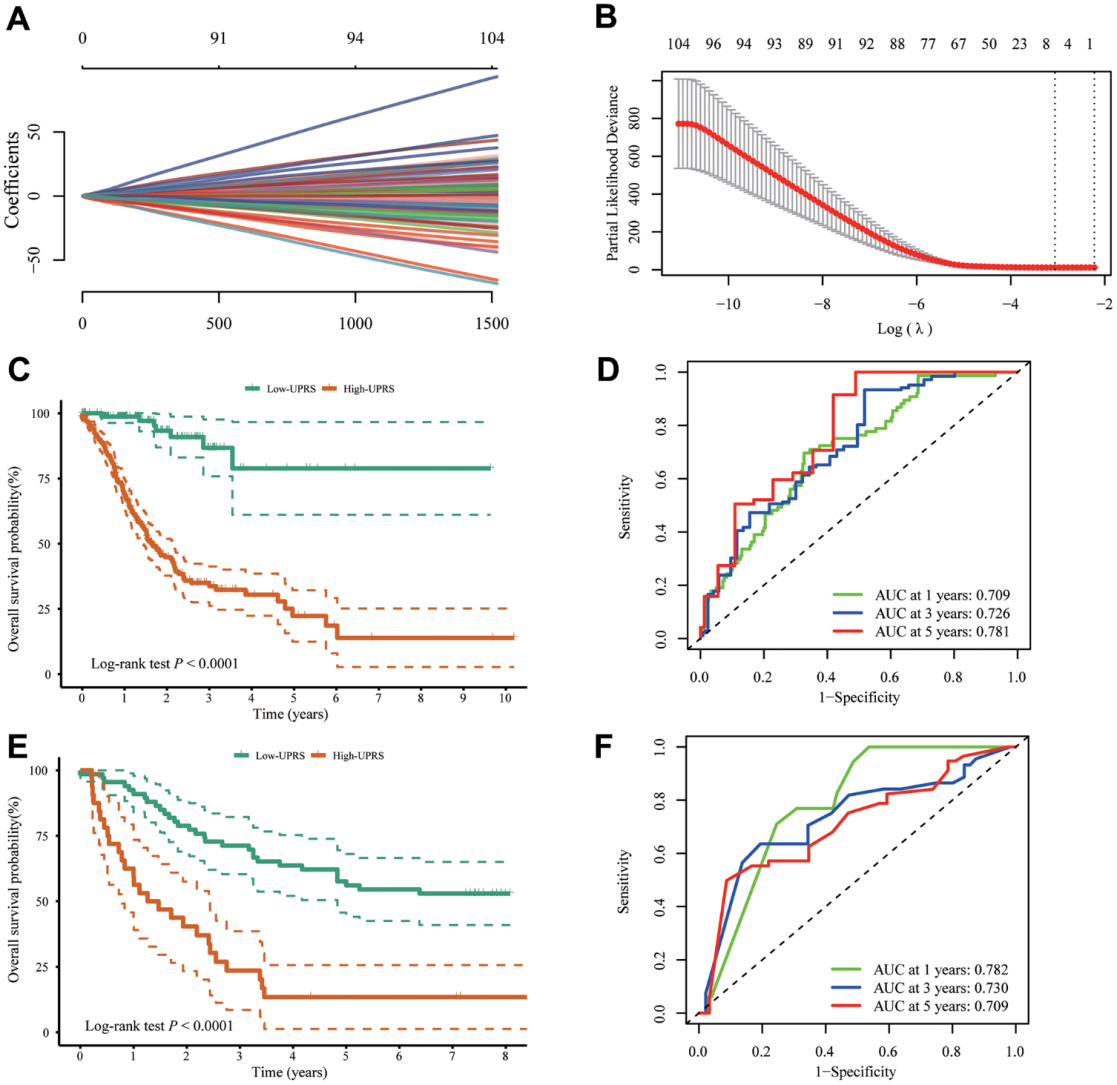
**Construction of UPR-related predictive signature for stomach cancer patients.** (**A**, **B**) LASSO Cox regression of differential expressed UPR-related genes in TCGA cohort. (**C**) The difference of overall survival between high- and low-UPRS patients in TCGA cohort. (**D**) ROC curve for the overall survival prediction in TCGA cohort. (**E**) The difference of overall survival between high- and low-UPRS patients in GSE13861 and GSE28541 cohort. (**F**) ROC curve for the overall survival prediction in GSE13861 and GSE28541 cohort.

In order to simplify the prognostic model, LASSO-Cox regression analysis was performed on UPR-related genes to establish UPR-related risk signature. Finally, 7 UPR-related genes were selected for prognostic model construction (Figure 7A, 7B). The risk score was calculated as follows: URPs = 0.2082 × EIF4E + 0.0556 × EXOSC1 - 0.1725 × IARS1 - 0.4039 × IGFBP1 + 0.0814 × SPCS1 + 0.1412 × TSPYL2 + 0.0951 × TUBB2A. Patients were divided into two risk groups based on their median scores. Kaplan-Meier curves consistently showed the patients at high risk had a worse OS than those at low risk (log-rank *P*<0.0001) (Figure 7C). In the analysis of survival prediction using the prognostic model, time-dependent receiver operating characteristic (ROC) curves were produced, with 1-, 3-, 5-year area under curves (AUCs) reaching 0.709, 0.726, 0.781 (Figure 7D). Based on the optimal cutoff value, patients in the GSE37023 cohort were also divided into high-risk and low-risk groups to assess the stability of the UPR-related signature. The high-risk group likewise demonstrated poorer overall survival (OS) compared to the low-risk group (log-rank *P*<0.0001) (Figure 7E). Likewise, the AUC of the predictive signature was 0.782 at 1 year, 0.730 at 3 years, and 0.709 at 5 years (Figure 7F).

**Figure 8:**
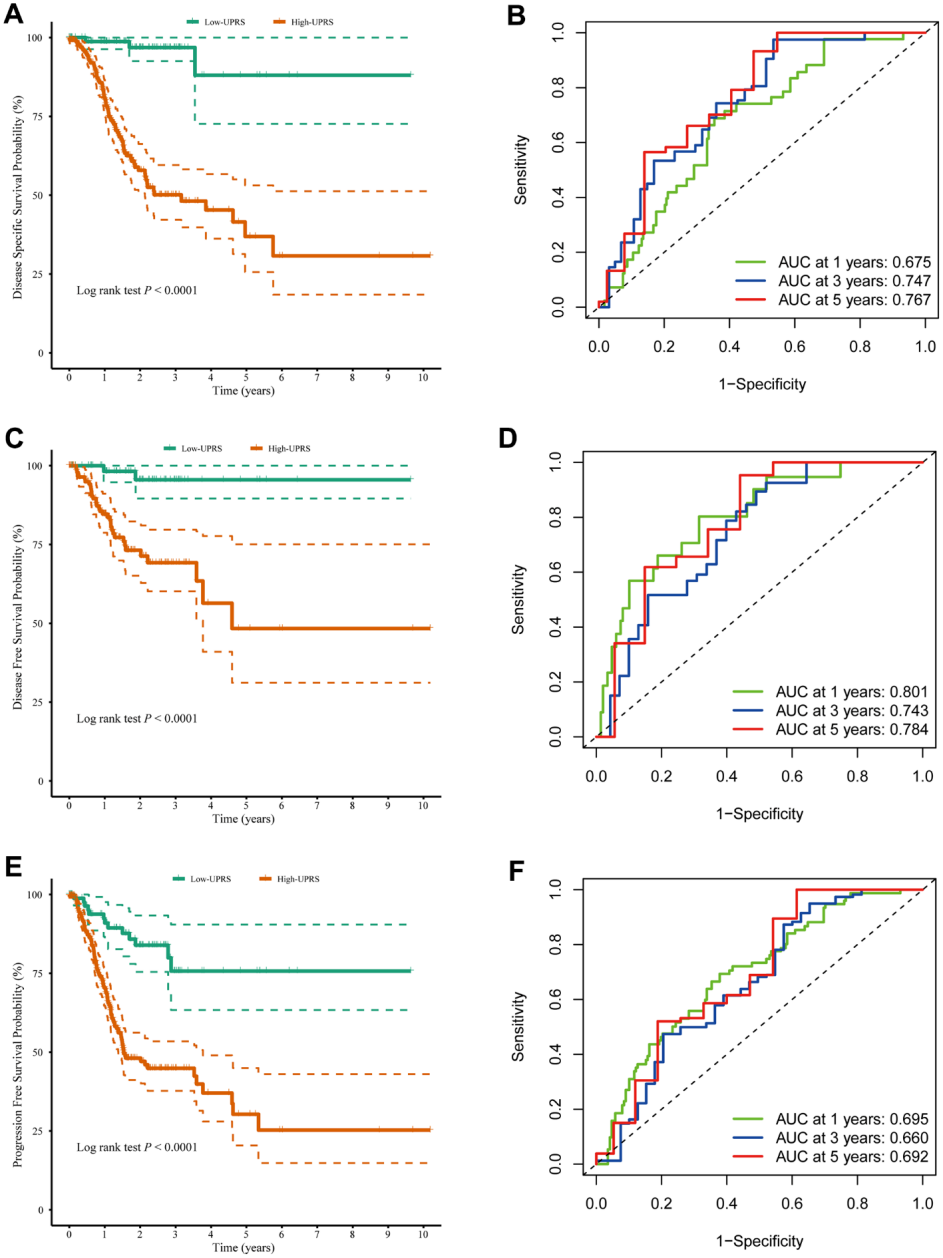
**The UPR-related predictive signature for stomach cancer patients.** (**A**) Disease-specific survival among different groups and (**B**) the ROC for disease-specific survival prediction. (**C**) Disease-free survival among different groups and (**D**) the ROC for disease-free survival prediction. (**E**) Progression-free survival among different groups and (**F**) the ROC for progression-free survival prediction.

In addition, the practical utility of the UPR signature when validated using other survival data from TCGA cohort. We observed that the high-risk group had lower disease specific survival compared to the low-risk group (log-rank p < 0.0001) (Figure 8A), with AUC values of 0.675, 0.747, and 0.767 at 1, 3, and 5 years, respectively (Figure 8B). Similarly, the high-risk group exhibited poorer disease-free survival than the low-risk group (log-rank p < 0.0001) (Figure 8C), with AUC values of 0.801, 0.743, and 0.784 at 1, 3, and 5 years (Figure 8D). Lastly, the high-risk group displayed lower progression free survival compared to the low-risk group (log-rank p < 0.0001) (Figure 8E), with AUC values of 0.695, 0.660, and 0.692 at 1, 3, and 5 years (Figure 8F). The clinical implications of these findings are significant. The TCGA stomach cancer prognostic UPR signature can be utilized as a valuable tool for risk stratification, enabling healthcare professionals to identify high-risk patients who may require closer monitoring, more aggressive treatment, or personalized therapeutic strategies. This risk assessment can lead to improved clinical decision-making and ultimately better patient outcomes. Furthermore, by understanding the underlying molecular mechanisms associated with different risk levels, researchers can potentially identify new therapeutic targets for the development of novel treatments for stomach cancer patients.

### Differences in immune cell infiltration levels between the URPS groups

In order to better investigate the differences in immune cell infiltration between high and low-risk groups, we employed multiple immune assessment algorithms.

**Figure 9:**
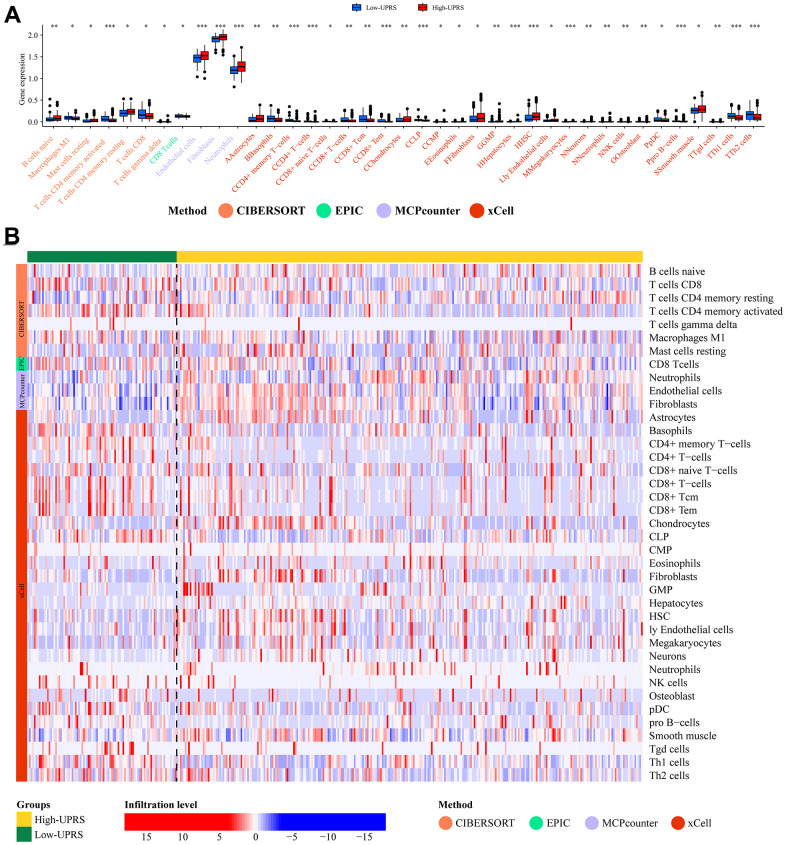
**Analysis of immune cell infiltration.** (**A**) The barplot and (**B**) heatmap showed the difference in immune cell infiltration between patients with high- and low-UPRS.

Figure 9A illustrates the difference in immune cell infiltration levels between high-URPs and low-URPs groups. The majority of immune cells exhibit higher expression levels in the low-URPs group, particularly M1 Macrophages (P<0.05), CD8+ T cells (P<0.05), CD4+ memory T cells (P<0.001), and CD8+ Tcm (P<0.01). Furthermore, the heat map in Figure 9B also reveals that the low-URPs group has increased immune cell expression. This finding suggests that patients in the low-risk group have stronger immune activity, which may contribute to a more favorable prognosis.

### Independent prognostic value of risk models and construction of nomogram

**Figure 10:**
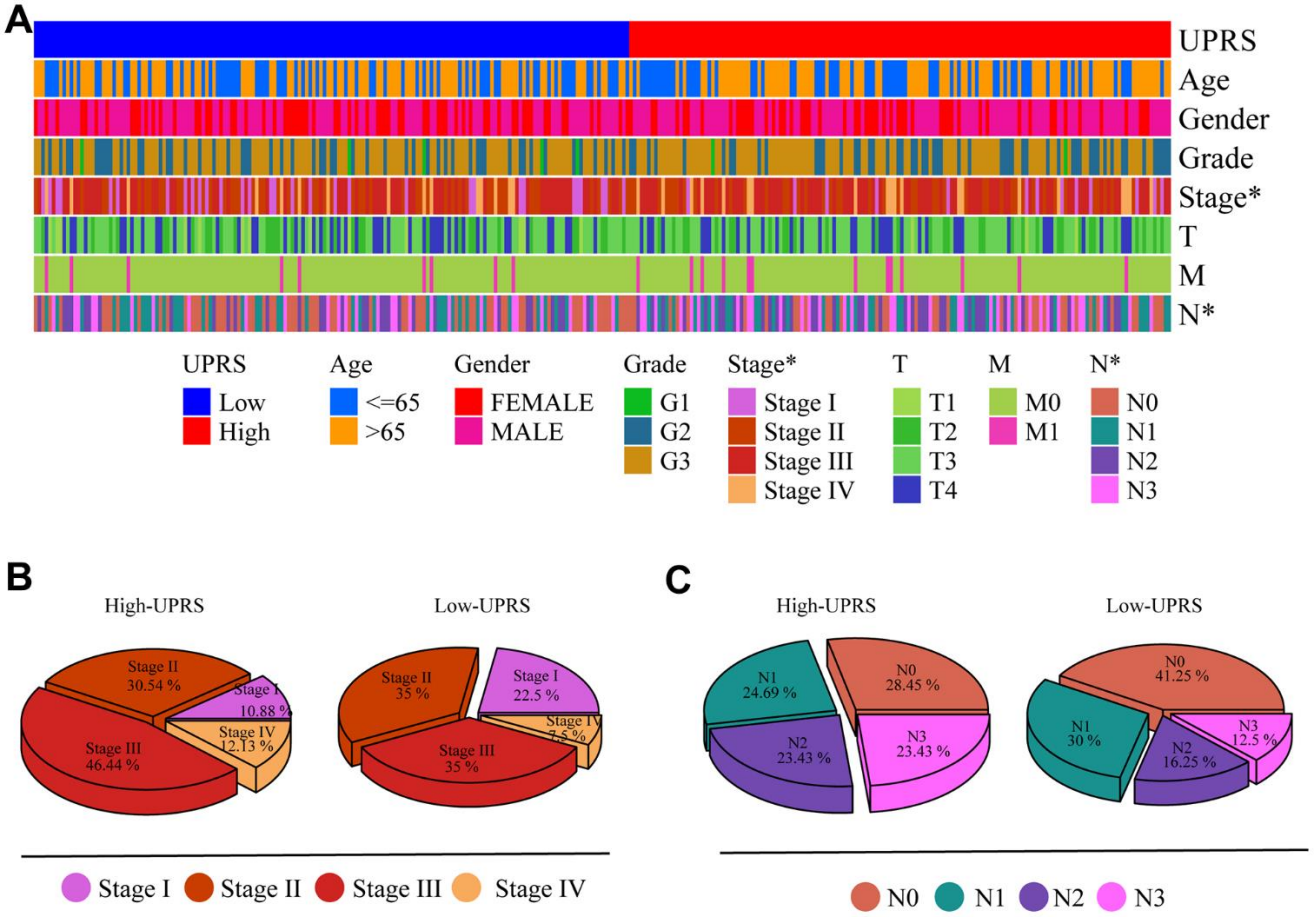
**Differences in clinical variables were analyzed in patients with high- and low-UPRS.** (**A**) Heat maps showed differences in clinical variables between patients with high- and low-UPRS. (**B**) The proportion of patients with high- and low-UPRS in each stage. (**C**) The proportion of patients with high- and low-UPRS in each N stage.

**Figure 11:**
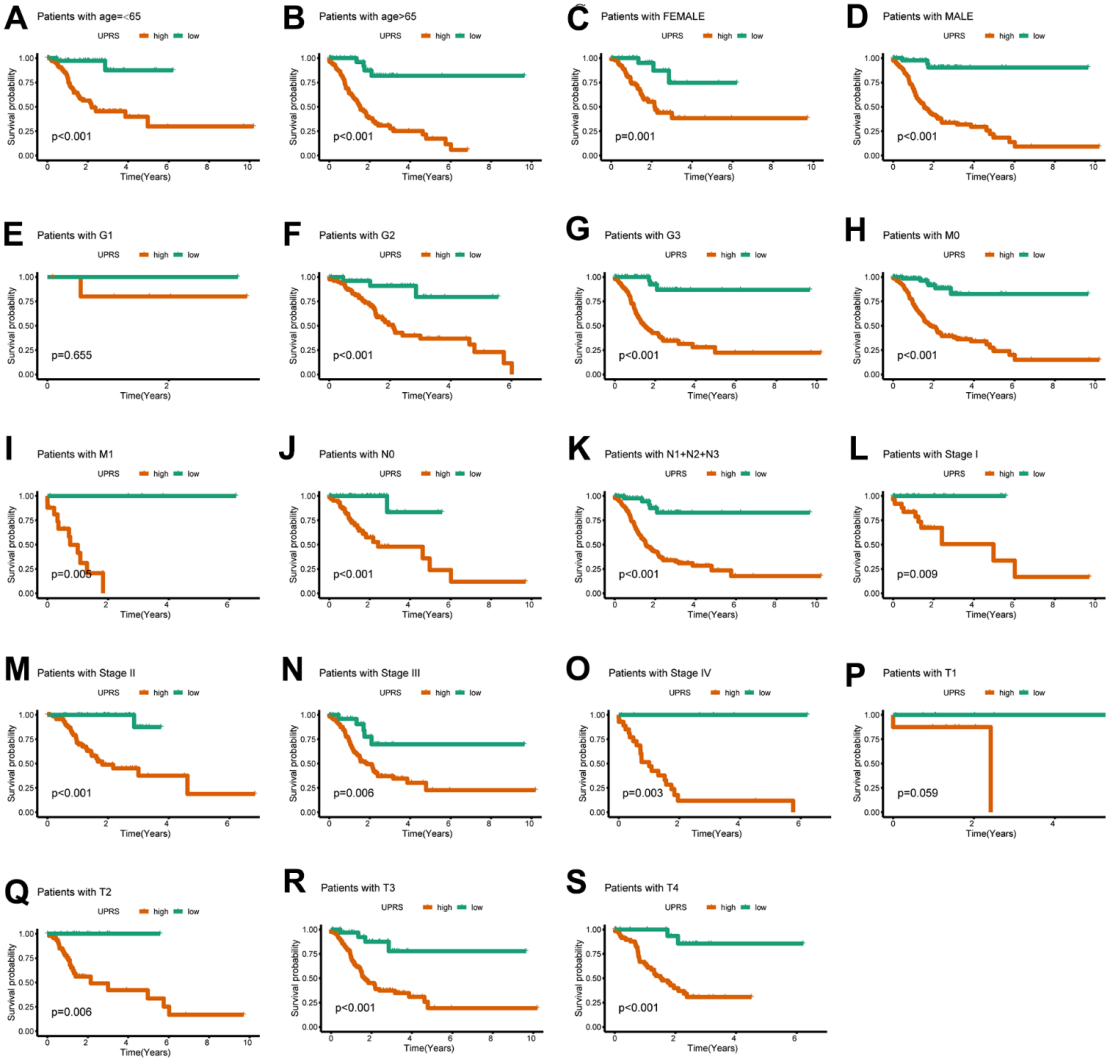
**The UPR-related predictive signature for stomach cancer patients in different clinical subgroups.** The KM curve for stomach cancer patients with (**A**) age≤65, (**B**) age>65, (**C**) Female, (**D**) Male, (**E**) Grade 1, (**F**) Grade 2, (**G**) Grade 3, (**H**) M0 stage, (**I**) M1 stage, (**J**) N0 stage, (**K**) N1+N2+N3 stage, (**L**) Stage I, (**M**) Stage II, (**N**) Stage III, (**O**) Stage IV, (**P**) T1 stage, (**Q**) T2 stage, (**R**) T3 stage, (**S**) T4 stage.

**Figure 12:**
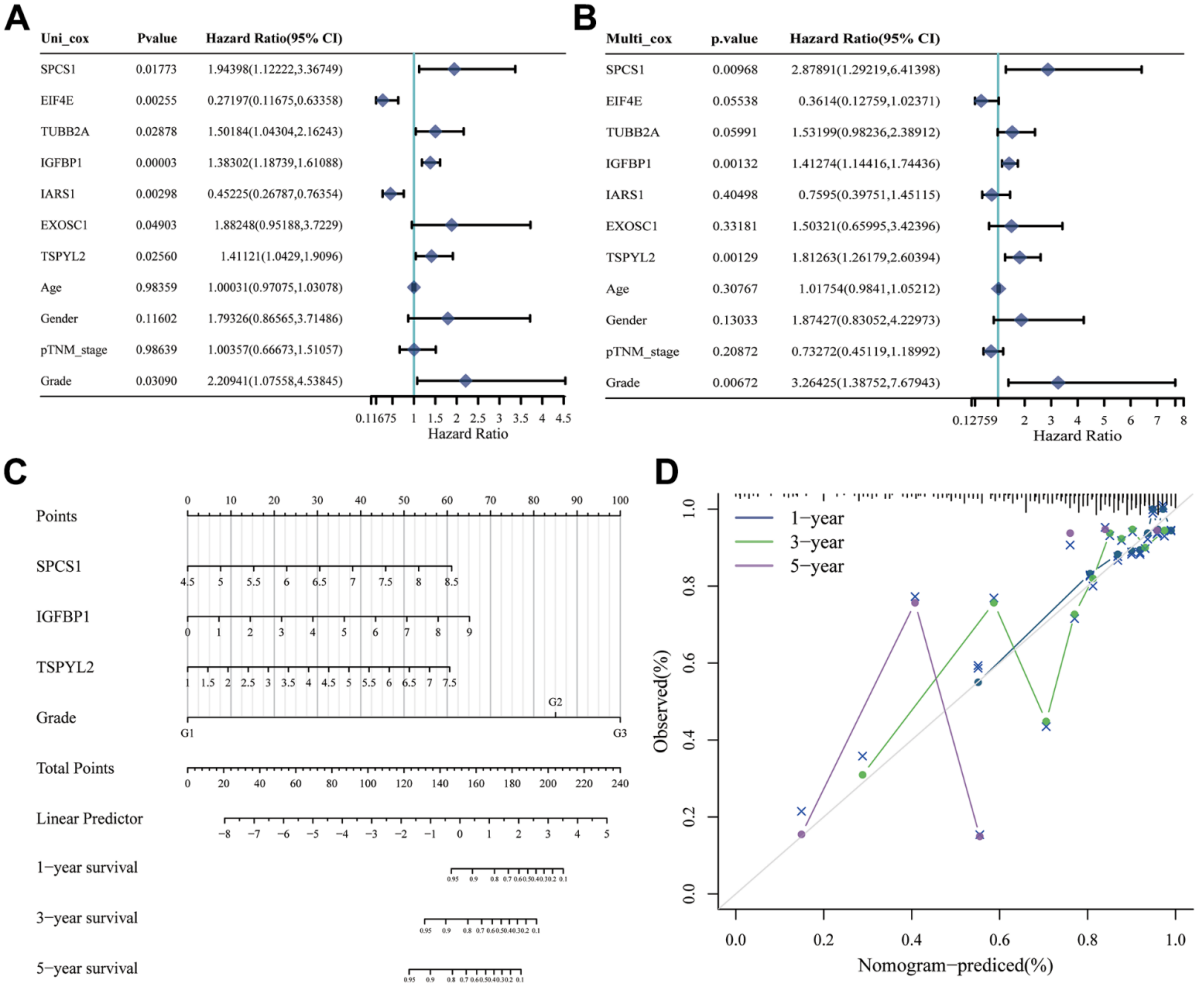
**Construction of nomogram based on independent predictors.** (**A**) Univariate Cox analysis and (**B**) multivariate Cox analysis were performed to figure out prognostic factor. (**C**) Nomogram was developed based on the results of multivariate Cox analysis. (**D**) Calibration curve of this nomogram indicated observed nomogram performance.

First, we analyzed the differences in various clinical factors (including age, gender, clinicopathological grade, clinicopathological stage, T stage, M stage, and N stage) between the low-URPs group and the high-URPs group in the TCGA cohort. We discovered that both stage and N stage were statistically different between the low-URPs and high-URPs groups (P<0.05) (Figure 10A). The composition of stage and N stage is illustrated in Figures 10B, 10C. Next, to broaden the risk model’s applicability, we performed validation for each stage of clinical factors, and the survival curve is presented in Figure 11. Furthermore, univariate and multivariate Cox regression analyses were conducted on the TCGA cohort to evaluate the risk model’s accuracy and determine if the risk score could serve as an independent prognostic factor for patient survival. Univariate Cox regression analysis revealed that age, stage, N stage and the genes of prognostic model were significantly associated with patient prognosis, after adjusting for other confounding factors, multivariate analysis indicated that age, grade, SPCS1, IGFBP1 and TSPYL2 were independent prognostic factors (Figure 12A, 12B).

Based on the TCGA cohort, we integrated clinical factors such as age, stage, N stage, SPCS1, IGFBP1 and TSPYL2 to construct a nomogram to enhance survival prediction for patients with stomach cancer (Figure 12C). Calibration plots for 1-, 3-, and 5-year overall survival (OS) demonstrated good agreement between nomogram predictions and actual observations (Figure 12D). Clinically, this research offers valuable insights into the prognostic factors of stomach cancer, which can help clinicians make more informed decisions regarding treatment strategies. By presenting the model in the form of a nomogram and calibration plots, we provide a more intuitive and user-friendly tool that can be easily implemented in clinical practice to improve patient outcomes.

### Pan-cancer analysis of IGFBP1

**Figure 13:**
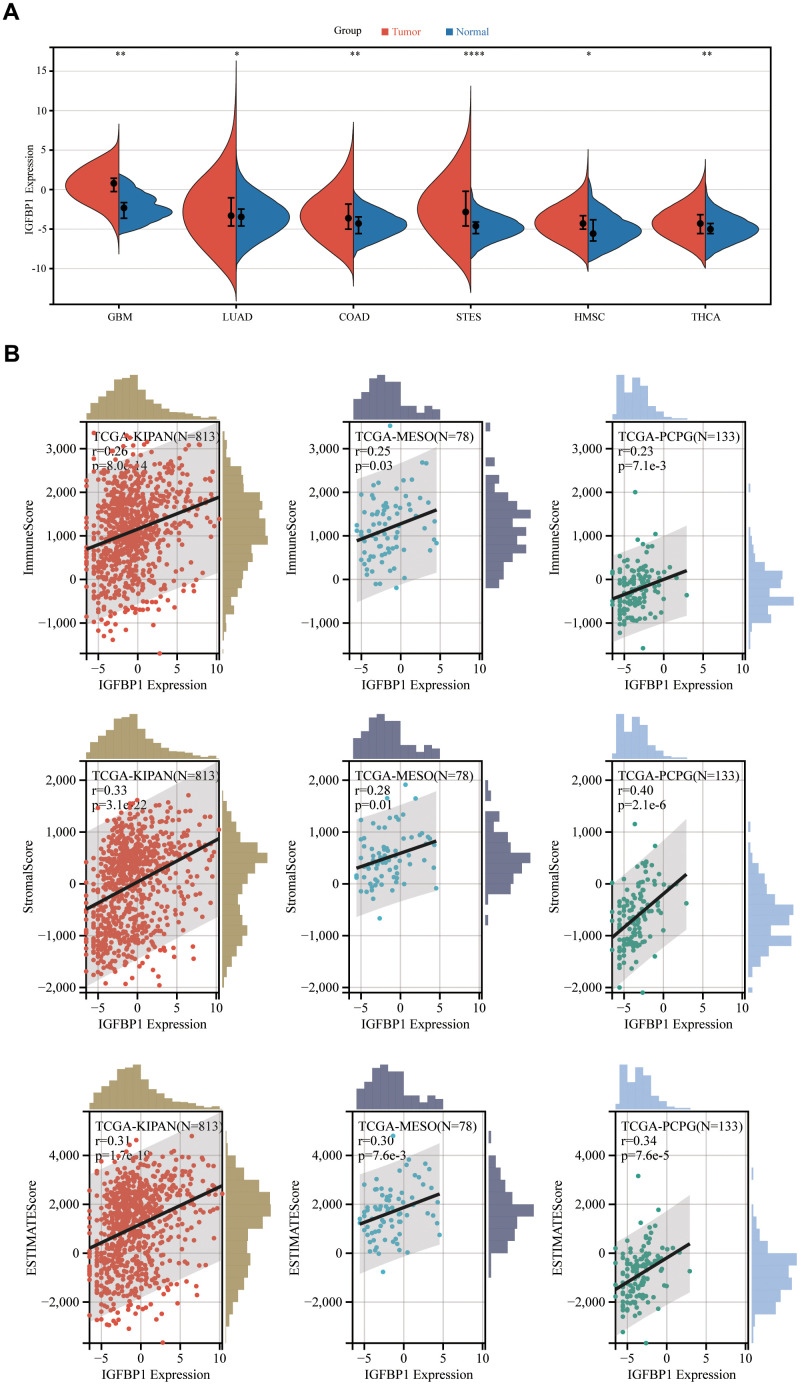
**Pan-cancer analysis of IGFBP1.** (**A**) The expression of IGFBP1 was compared between tumor tissue and normal tissue in other different types of cancer. (**B**) Association of IGFBP1 expression and immune score, stromal score or tumor purity was evaluated using ESTIMATE analysis. GBM, glioblastoma multiforme; LUAD, lung adenocarcinoma; COAD, colon adenocarcinoma; STES, stomach and esophageal carcinoma; HNSC, head and neck squamous cell carcinoma; THCA, thyroid carcinoma; KIPAN, pan-kidney carcinoma; MESO, mesothelioma; PCPG, pheochromocytoma and paraganglioma. *P < 0.05, **P < 0.01, ***P < 0.001, ****P < 0.0001.

We have constructed a prognostic scoring model comprising seven genes, with IGFBP1 having the highest absolute coefficient value. Consequently, we consider IGFBP1 to be the key gene in our model. Furthermore, we aim to investigate the expression of this gene in other types of tumors and its relationship with the immune microenvironment. Then, other pan-cancer analysis revealed that the expression of IGFBP1 in tumor tissues was significantly higher than that in normal tissues in TCGA-GBM, TCGA-LUAD, TCGA-COAD, TCGA-STES, TCGA-HMSC, and TCGA-THCA (Figure 13A). Additionally, IGFBP1 expression was positively correlated with ImmuneScore, StromalScore, and ESTIMATEScore in TCGA-KIPAN, TCGA-MESO, and TCGA-PCPG (Figure 13B). These findings demonstrate the potential role of IGFBP1 as a biomarker for tumor progression and the tumor microenvironment. Understanding the involvement of IGFBP1 in various cancers and its association with immune and stromal components may provide valuable insights for the development of novel therapeutic strategies, leading to improved patient outcomes.

### IGFBP1 promotes the malignant phenotype of stomach cancer cells

**Figure 14:**
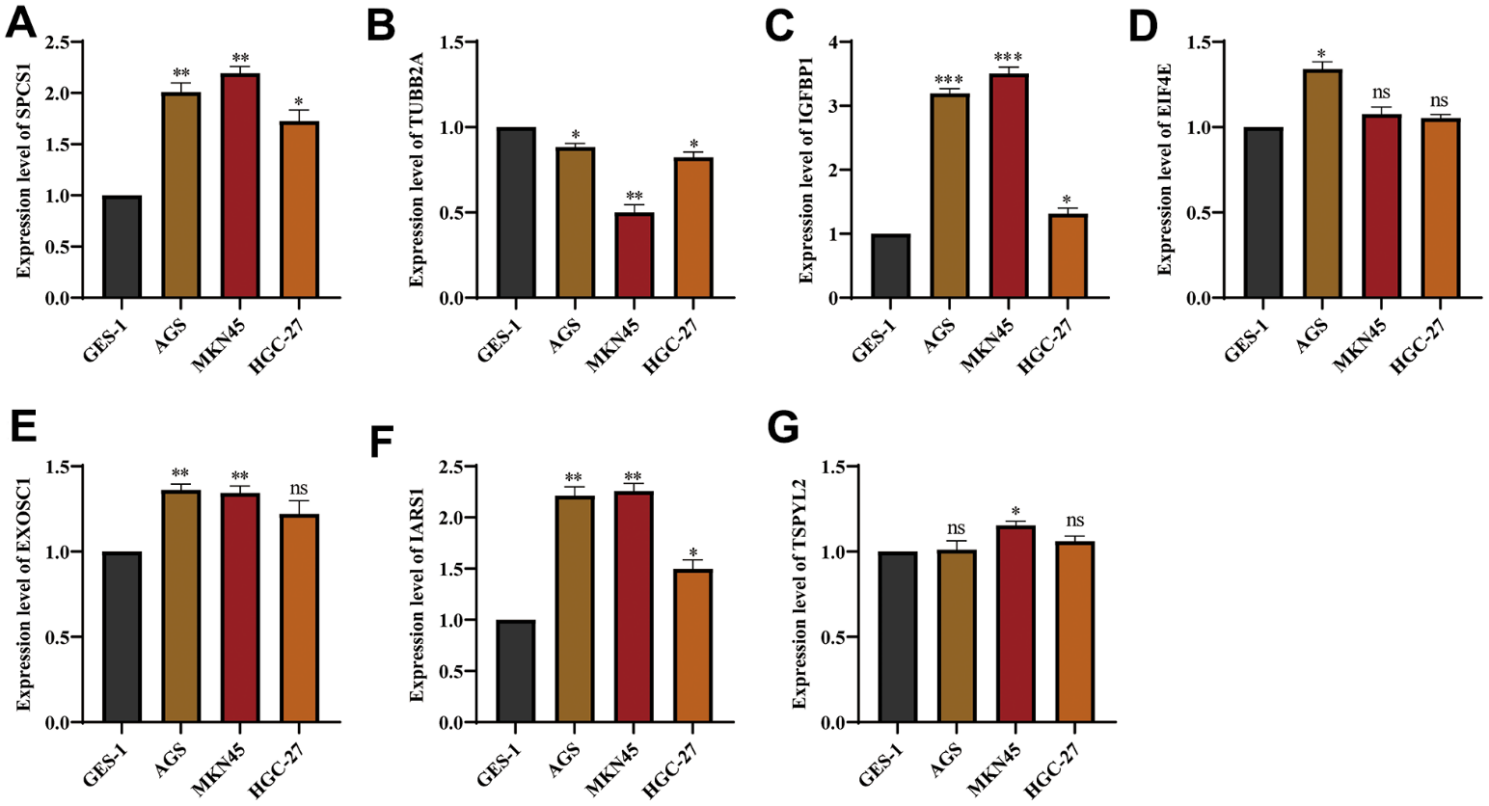
**The expression of genes from UPR-related prognostic model in stomach cancer cell lines.** The mRNA expression level of (**A**) SPCS1, (**B**) TUBB2A, (**C**) IGFBP1, (**D**) EIF4E, (**E**) EXOSC1, (**F**) IARS1 and (**G**) TSPYL2 in GES-1, AGS and MKN45 cell lines. ns, not significant. *P < 0.05, **P < 0.01, ***P < 0.001.

We used three cell lines GES-1, AGS, MKN45 and HGC-27 to verify the expression of genes from UPR-related prognostic model. The expression of EIF4E, EXOSC1, IARS1, IGFBP1, and SPCS1 was found to be upregulated in stomach cancer cell lines, while TSPYL2 and TUBB2A showed downregulated expression (Figure 14A–14G). Supplementary Table 2 contains a list of all primer sequences. Notably, IGFBP1 demonstrated significant differences in expression among the stomach cancer cell lines. As a result, we further investigated the functionality of IGFBP1.

**Figure 15:**
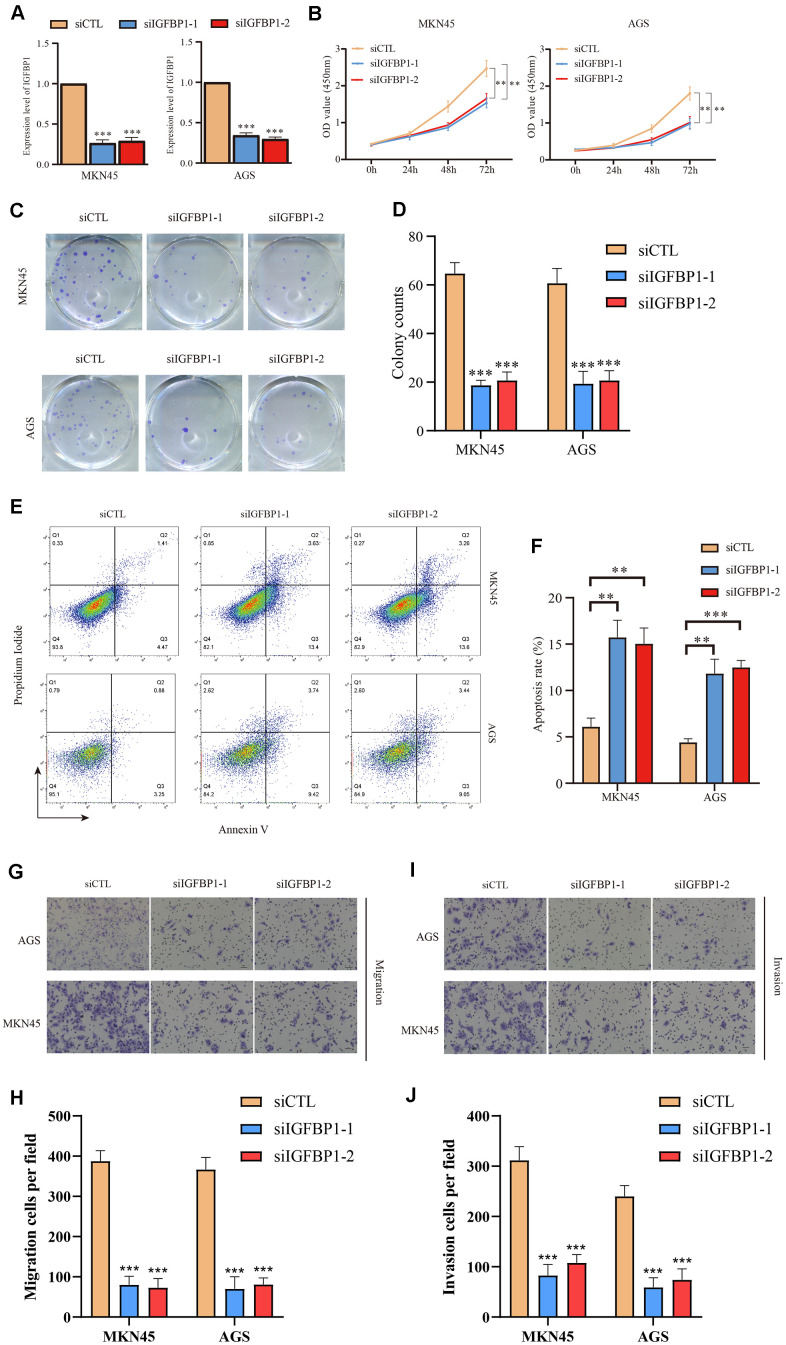
**Functional validation of IGFBP1 in AGS and MKN45 cell lines.** (**A**) The knockdown efficiency of IGFBP1 was tested by qPCR in MKN45 and AGS cell lines. (**B**) The proliferation efficiency of MKN45 and AGS cell lines with IGFBP1 knockdown by CCK8 assay. (**C**) The proliferation efficiency of MKN45 and AGS cell lines with IGFBP1 knockdown by cell cloning formation. (**D**) The statistical analysis of (**C**). (**E**) The apoptosis of MKN45 and AGS cell lines with IGFBP1 knockdown. (**F**) The statistical analysis of (**E**). (**G**) The migration efficiency of MKN45 and AGS cell lines with IGFBP1 knockdown. (**H**) The statistical analysis of (**G**). (**I**) The invasion efficiency of MKN45 and AGS cell lines with IGFBP1 knockdown. (**J**) The statistical analysis of (**I**).

Then, using the stomach cancer cell lines MKN45 and AGS, we performed loss-of-function experiments and silenced expression of *IGFBP1* to investigate its biological function. The efficiency of two siRNAs (siIGFBP1-1 and siIGFBP1-2) knockdown of IGFBP1 (Figure 15A) were confirmed by RT-qPCR, and the sequences of both siRNAs are presented in Supplementary Table 3. *In vitro*, the knockdown of IGFBP1 resulted in a decrease of the proliferation of MKN45 and AGS cells as measured by the CCK8 assay (Figure 15B) and clonal formation assay (Figure 15C, 15D). The proportion of apoptotic cells increased significantly (MKN45, *P*<0.01; AGS, *P*<0.01) by knockdown of *IGFBP1,* indicating that IGFBP1 significantly decreased the antiapoptotic ability of the MKN45 and AGS cells (Figure 15E, 15F). The knockdown of *IGFBP1* significantly decreased migration and invasion of both MKN45 and AGS cells in the Transwell migration and invasion assay (MKN45, *P*<0.001; AGS, *P*<0.001) (Figure 15G–15J). It has been demonstrated *in vitro* experiment that the *IGFBP1* promotes the malignant phenotype of stomach cancer, including proliferation, invasion, and migration. In view of this, IGFBP1 is expected to become a new target for the treatment of stomach cancer.

### Mechanistic insights into the role of IGFBP1 in the unfolded protein response of gastric cancer

**Figure 16:**
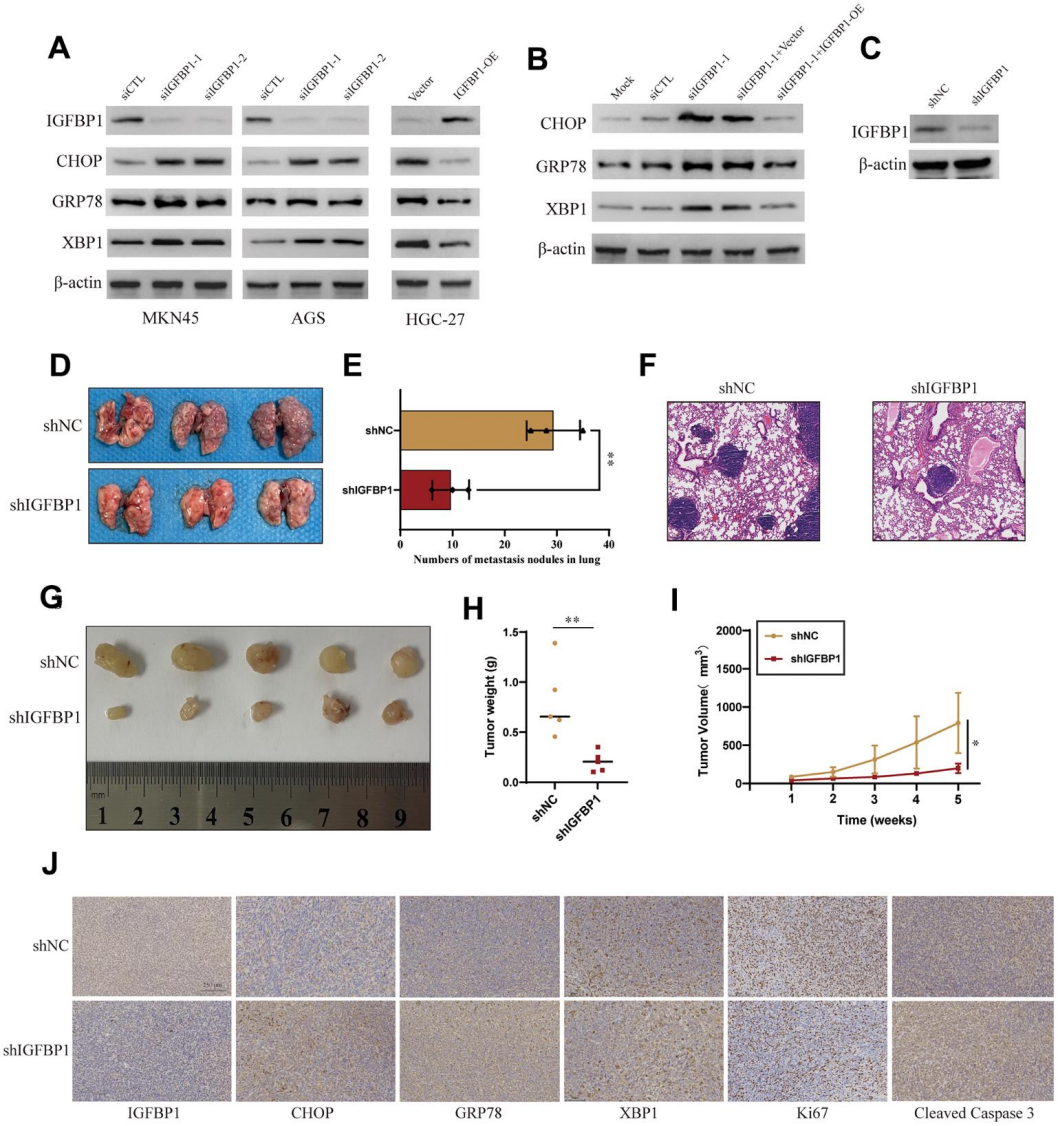
**Validation of the role of IGFBP1 in the unfolded protein response of gastric cancer.** (**A**) MKN45 and AGS cells were transfected with siIGFBP1 and overexpressed IGFBP1 in HGC-27 cells. (**B**) Protein expression changes of CHOP, GRP78, XBP1 after IGFBP1 supplementation in MKN45 cells. (**C**) Verify knock down efficiency of shIGFBP1. (**D**) Anatomical images of lung metastases 4 weeks after caudal vein inoculation with gastric cancer cells. Lung metastasis was inhibited in cancer cells transfected with shIGFBP1 compared to shNC. (**E**) Statistical histogram of pulmonary metastatic nodules. (**F**) H&E staining in the pathology of lung metastases. (**G**) Images of xenografts dissected from nude mice 5 weeks after subcutaneous injection. (**H**) Graph showing the average tumor weight of the resected tumor. (**I**) Tumor volume curve showing tumor growth. (**J**) Representative IHC images of the shIGFBP1 and shNC were differentially expressed CHOP, GRP78, XBP1, Ki67 and Cleaved Caspase 3. *P < 0.05, **P < 0.01.

In gastric cancer cell lines AGS and MKN45, IGFBP1 was knocked down for three days, followed by treatment with 2 μg/ml Tunicamycin to induce ER stress, for a duration of 24 hours [20]. We monitored the expression of UPR biomarkers (CHOP, GRP78, XBP1). Western blot analysis indicated that, upon IGFBP1 knockdown in AGS and MKN45 cells, there was a notable upregulation of CHOP, GRP78, and XBP1 compared to the NC group, suggesting that cells under ER stress require a more robust UPR activation to adapt to the ER condition. To validate this observation, we overexpressed IGFBP1 in another gastric cancer cell line with relatively low IGFBP1 expression, HGC-27, and treated with Tunicamycin to induce ER stress. It was found that the UPR triggered by ER stress was not as intense with IGFBP1 overexpression (Figure 16A). Furthermore, upon re-expressing IGFBP1 in IGFBP1-knockdown MKN45cells, the UPR response to ER stress was alleviated after IGFBP1 supplementation (Figure 16B). These results confirm that IGFBP1 facilitates gastric cancer adaptation to ER stress.

Stable IGFBP1-knockdown MKN45cell lines were established, and the knockdown efficiency of IGFBP1 was confirmed via Western blot (Figure 16C). To further investigate the impact of IGFBP1 on the metastatic capability of gastric cancer, we injected the blank control group (shNC) and the stable IGFBP1-knockdown cell line (shIGFBP1 group) into nude mice via tail vein. After four weeks, lung metastasis was significantly more pronounced in mice from the shIGFBP1 group compared to the shNC group (Figure 16D, 16E). H&E staining of mouse lung tissue slices revealed multiple tumor nodules and dense cancer cells. In contrast, the experimental group showed fewer nodules and cancer cells (Figure 16F), suggesting that IGFBP1 promotes gastric cancer metastasis.

Furthermore, subcutaneous xenografts in nude mice using shNC and shIGFBP1 cells, and regular measurements of tumor volume, indicated that tumor growth in terms of average volume and weight was significantly inhibited in the shIGFBP1 group compared to the control group (Figure 16G–16I). IHC images of tumor slices showed that the shIGFBP1 group exhibited higher expression of CHOP, GRP78, XBP1 (Figure 16J). Additionally, Ki-67 and Cleaved Caspase 3 staining images revealed that the tumor tissue in the shIGFBP1 group had the lowest level of Ki-67 proliferation and the highest level of apoptosis (Figure 16J). These animal experiments demonstrate that IGFBP1 not only enhances ER adaptability but also promotes gastric cancer progression, thereby nominating IGFBP1 as a potential therapeutic target for gastric cancer.

## DISCUSSION

Stomach cancer is characterized by a high incidence and mortality rate, posing a significant health challenge worldwide. Despite advances in medical research, stomach cancer prognosis remains difficult to predict. The development of reliable prognostic indicators for stomach cancer is crucial for improving patient outcomes and guiding treatment strategies. Research suggests that the unfolded protein response (UPR) may be one of the novel mechanisms involved in regulating stomach cancer progression [21].

Herein, in this study, we developed a classifier based on UPR-related genes, which effectively stratified stomach cancer patients into two distinct molecular subtypes. Further analysis revealed that these subtypes exhibited significant differences in prognosis, immune microenvironment infiltration, and chemosensitivity. Subsequently, we constructed a prognostic scoring formula using seven UPR-associated genes, which effectively predicted the prognosis of stomach cancer patients. Finally, we identified IGFBP1 as a crucial factor in the prognostic model and verified its role in promoting the malignant phenotype of stomach cancer cells through functional experiments.

The unfolded protein response (UPR) operates as an adaptive mechanism during cancer progression, modulating mechanisms that trigger cell transformation, enhance survival, and adjust metabolic status [22]. The initiation of the UPR in tumor cells, especially when subjected to high levels of endoplasmic reticulum (ER) stress, plays an important survival strategy [23]. When these stress response mechanisms fail to fully correct the protein accumulation problem, the UPR further promotes tumor cells to initiate the process of autophagy, an intracellular recycling mechanism for degrading and recycling defective cellular components, including damaged organelles and protein aggregates [24–26]. This process not only helps to maintain the stability of the intracellular environment, but also reduces the risk of cell death due to accumulated damage. Previous research has proved that UPR-related IRE1/XBP1 signaling pathway plays a role in tumor progression, with high expression of XBP1s protein correlating with poor prognosis in glioblastoma, triple-negative breast cancer, and pre-B acute lymphoblastic leukemia [27–29]. Therefore, UPR is closely related to the progression of multiple tumors. In addition, studies have shown that GPCRs modulate UPR signaling via ERS sensors, IRE1α, PERK, and ATF6, to support cancer cell survival and inhibit cell death [30]. By regulating downstream signaling pathways such as NF-κB, PI3K/AKT, TGF-β and Wnt/β-catenin, GPCRs also upregulate mesenchymal transcription factors including Snail, ZEB, and Twist superfamilies which regulate cell polarity, cytoskeleton remodeling, migration, and invasion [31, 32]. These pathways play a critical role in various pathological conditions in humans, including cancer, by regulating a number of key processes involved in tumor formation and progression [33, 34].

In addition to UPR affecting the development of the tumor itself, he can also affect the sensitivity of the tumor to cisplatin therapy, as shown by a study that showed that the unfolded protein response (UPR) plays a key role in cisplatin-based drug resistance. Drugs such as cisplatin, carboplatin, and oxaliplatin are inactivated by covalent binding to glutathione, which reduces the binding of the drug to its target and facilitates drug efflux through ABC transporter proteins, leading to resistance [35–37]. In particular, under chemotherapeutic stress, the UPR is activated through the PERK pathway, which upregulates the expression of ATF4 and NRF2, thereby enhancing the expression of antioxidant genes (e.g., GST, GCLC) [38]. These gene products promote drug binding to glutathione, which further leads to inactivation of multiple chemotherapeutic agents. Thus, high levels of glutathione are closely associated with cisplatin resistance. Furthermore, it has been found that in order to attenuate cisplatin-induced acute kidney injury, p38 MAPK mediation can be inhibited by 7-hydroxycoumarin-β-D-glucuronide (7-HCG) [39]. Our study reveals that clustering stomach cancer patients based on UPR-related gene signatures effectively classifies them into distinct molecular subtypes with different prognoses. The analysis showed that patients in Cluster 1 molecular subtype had a less favorable overall survival, disease-free survival, progression-free survival, and disease-specific survival probability compared to those in Cluster 2 molecular subtype. Furthermore, differentially expressed genes between the two clusters were significantly enriched in cancer progression-related biological processes and pathways, such as the TGF-β and NF-κB signaling pathways as well. In accordance with previous research findings, our study demonstrated that the TGF-β and NF-κB pathways are indeed classical UPR pathways involved in tumor development in accordance with previous research findings, our study confirms that the TGF-β and NF-κB pathways are indeed classical UPR pathways involved in stomach cancer development.

Prognostic signature constructed in this study consisted of 7 UPR-related genes, among which IGFBP1 showed the highest positive correlation coefficient. IGFBP1 as a protein prolonging the half-life of the insulin-like growth factors (IGFs) by combination, is induced during ER stress via activating transcription factor 4 (ATF4) expression [40]. In this study, we found that IGFBP1 knockdown in gastric cancer cells AGS and MKN45 amplifies the UPR under ER stress, suggesting that IGFBP1 plays a critical role in ER stress adaptability. Conversely, overexpressing IGFBP1 dampens this response. *In vivo* experiments confirm that reduced IGFBP1 expression correlates with decreased metastasis and tumor growth, underscoring its significance in cancer progression and as a target for therapeutic intervention.

Increased secretion of IGFBP1 helps cells modulate cell metabolism and maintain adaptive response under ER stress. IGFBP1 may inhibit IGFs activity by attenuating the binding of IGFs to their receptors [41], or potentiate IGFs function by facilitating the interaction of IGFs with receptors or binding to cell membranes through IGFBP1’s Arg-Gly-Asp sequence [42]. Post-translational modifications of IGFBP1 may affect the regulation of IGFBP1 on IGFs [43]. However, complex regulatory network among IGFs and IGFBPs is still not elucidated. Also, other IGF-independent effects of IGFBP1 were found to regulate cell migration and growth by interaction with extracellular or intracellular partners when faced with ER stress [44]. Taken together, this study we conducted *in vitro* found that IGFBP1 is capable of proliferating, invasive and migrating stomach cancer cells in a proliferation-like manner.

## CONCLUSIONS

In summary, UPR-related gene classifier and risk signature were constructed for survival prediction of patients with stomach cancer. The patients in Cluster 1 molecular subtype tended to be characterized as short survival time, low chemotherapy sensitivities, and low tumor-killing immune cells infiltration. Next, we developed a robust prognostic prediction signature based on 7 UPR-related genes, and examined the relationship between this risk model and the prognosis of stomach cancer patients. As a key component of the UPR prognostic signature, IGFBP1 serves as an independent prognostic factor for poor stomach cancer outcomes. We have confirmed that IGFBP1 promotes the malignant phenotype of stomach cancer, including proliferation, anti-apoptosis, invasion, and migration. *In vitro* and *in vivo* experiments validate that IGFBP1 facilitates enhanced adaptation of stomach cancer cells to endoplasmic reticulum contingency. IGFBP1 is expected to become an important therapeutic target for stomach cancer.

## Supplementary Material

Supplementary Tables
