# Supplementary material for: Unraveling the unfolded protein response signature: implications for tumor immune microenvironment heterogeneity and clinical prognosis in stomach cancer
Source: Aging (Albany NY). 2024 May 2;16(9):7818–44. doi: 10.18632/aging.205784 (PMC11132010; doi:10.18632/aging.205784)
Supplement: Supplementary Tables [file aging-16-205784-s001.pdf]

SUPPLEMENTARY TABLES

Supplementary Table 1. Clinical information of patients with stomach cancer in this study.

Cohort	TCGA	GSE13861	GSE28541
**Number of patients**	n=375	n=60	n=40
**Age (Mean±SD)**	65.83±10.65	60.87±11.78	58.40±13.06
**Follow up time (Mean±SD) (months)**	18.84±17.90	65.73±32.75	24.58±24.73
**Follow up status**			
Alive	228 (60.8%)	34 (56.7%)	8 (20.0%)
Dead	147 (39.2%)	26 (43.3%)	32 (80.0%)
**Gender**			
Male	241 (64.3%)	42 (70.0%)	27 (67.5%)
Female	134 (35.7%)	18 (30.0%)	13 (32.5%)
**Clinical stage**			
Stage I	53 (14.1%)	-	1 (2.5%)
Stage II	111 (29.6%)	-	6 (15%)
Stage III	150 (40.0%)	-	12 (30.0%)
Stage IV	38 (10.1%)	-	21 (52.5%)
Unknown	23 (6.1%)	-	-
**Grade**			
G1	10 (2.7%)	-	-
G2	137 (36.5%)	-	-
G3	219 (58.4%)	-	-
GX	9 (2.4%)	-	-
**T stage**			
T1	19 (5.1%)	-	-
T2	80 (21.3%)	-	-
T3	168 (44.8%)	-	-
T4	100 (26.7%)	-	-
TX	8 (2.1%)	-	-
Unknown	-	-	-
**M stage**			
M0	330 (88.0%)	52 (86.6%)	-
M1	25 (6.7%)	4 (6.7%)	-
MX	20 (5.3%)	-	-
Unknown	-	4 (6.7%)	-
**N stage**			
N0	111 (29.6%)	-	-
N1	97 (25.9%)	-	-
N2	75 (20.0%)	-	-
N3	74 (19.7%)	-	-
NX	16 (4.3%)	-	-
Unknown	2 (0.5%)	-	-

Supplementary Table 2. Summary of primers of each gene.

id	Primer	direction	Sequence (5' > 3')
EIF4E	Forward	primer	5'-GAAACCACCCCTACTCCTAATCC-3'
	Reverse	primer	5'-AGAGTGCCCATCTGTTCTGTA-3'
EXOSC1	Forward	primer	5'-GCACAGTCCAACTACCTGCT-3'
	Reverse	primer	5'-GTGTGGGTCTTAGGGCACTG-3'
IARS1	Forward	primer	5'-TTGCACCGCATCTCTGAAGT-3'
	Reverse	primer	5'-TGCCCTCGGCAATGAAATCT-3'
IGFBP1	Forward	primer	5'-AGGCACAGGAGACATCAGGA-3'
	Reverse	primer	5'-CCATTCCAAGGGTAGACGCA-3'
SPCS1	Forward	primer	5'-ATCTACGGGTACGTGGCTGA-3'
	Reverse	primer	5'-AACCACTTGAGAGGATGCCG-3'
TSPYL2	Forward	primer	5'-CCCAGAGGCTGACAGGATTG-3'
	Reverse	primer	5'-CTCACATCTGCCCCTGGTTT-3'
TUBB2A	Forward	primer	5'-CGCGCACCGCTCCGA-3'
	Reverse	primer	5'-CTGATGACCTCCCAAAACTTGGC-3'
β-actin	Forward	primer	5'-GGACCTGACTGACTACCTCAT-3'
	Reverse	primer	5'-CGTAGCACAGCTTCTCCTTAAT-3'

Supplementary Table 3. The sequence of siIGFBP1.

id	Sequence (5' > 3')
siCTL	5'-CCUAAGGUUAAGUCGCCCUCG-3'
siIGFBP1-1	5'-CAGGAGAAGAAAUUUCCAAAU-3'
siIGFBP1-2	5'-CCUGGAUAAUUUCCAUCUGAU-3'

siCTL, siRNA control.
